# DNA Methylation Analysis: Choosing the Right Method

**DOI:** 10.3390/biology5010003

**Published:** 2016-01-06

**Authors:** Sergey Kurdyukov, Martyn Bullock

**Affiliations:** 1 Genomics Core facility, Kolling Institute of Medical Research, University of Sydney, Sydney 2065, Australia; 2 Cancer Genetics Laboratory, Kolling Institute of Medical Research, University of Sydney, Sydney 2065, Australia; martyn.bullock@sydney.edu.au

**Keywords:** DNA methylation, 5-methylcytosine, CpG islands, epigenetics, next generation sequencing

## Abstract

In the burgeoning field of epigenetics, there are several methods available to determine the methylation status of DNA samples. However, choosing the method that is best suited to answering a particular biological question still proves to be a difficult task. This review aims to provide biologists, particularly those new to the field of epigenetics, with a simple algorithm to help guide them in the selection of the most appropriate assay to meet their research needs. First of all, we have separated all methods into two categories: those that are used for: (1) the discovery of unknown epigenetic changes; and (2) the assessment of DNA methylation within particular regulatory regions/genes of interest. The techniques are then scrutinized and ranked according to their robustness, high throughput capabilities and cost. This review includes the majority of methods available to date, but with a particular focus on commercially available kits or other simple and straightforward solutions that have proven to be useful.

## 1. Introduction

DNA methylation in vertebrates is characterized by the addition of a methyl or hydroxymethyl group to the C5 position of cytosine, which occurs mainly in the context of CG dinucleotides. Non-CpG methylation in a CHH and CHG context (where H = A, C or T) exist in embryonic stem cells and in plants. The aim of this review is to inform biologists studying DNA methylation of the pros and cons of the different assays currently available; allowing them to make an informed choice when deciding the technique that would best suit their research needs.

Most importantly, the method of choice should deliver an unbiased answer to the biological question being asked by the researcher. However, there are several other key factors that must be considered when choosing a method for DNA methylation analysis:

- The aims of the study (e.g., the discovery of *de novo* epigenetic changes or the investigation of known methylation sites of specific genes of interest);
- The amount and quality of the DNA sample(s) (e.g., Formalin-Fixed Paraffin-Embedded (FFPE) *versus* frozen tissue-derived DNA);
- The sensitivity and specificity requirements of the study;
- The robustness and simplicity of the method;
- The availability of bioinformatics software for analysis and interpretation of the data;
- The availability of specialized equipment and reagents;
- Cost.

**Figure 1:**
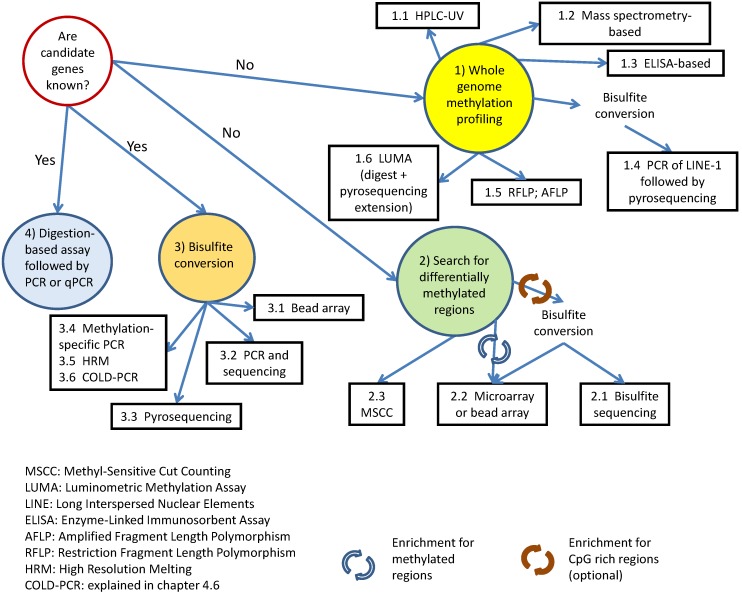
Algorithm for choosing a suitable method for DNA methylation analysis.

**Figure 2:**
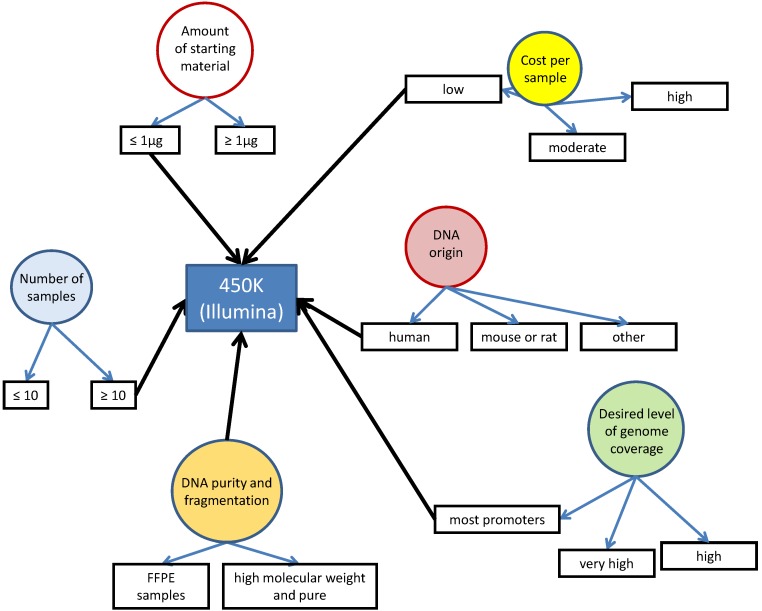
An example of how the algorithm can be applied.

Figure 1 provides a graphical guide for choosing the right method for a specific project using a simple algorithm. The following subsections of the review will describe each method, as well as highlight their pros and cons. Furthermore, an example application of the proposed algorithm is illustrated in Figure 2. Not all possible techniques that exist will be covered in this review, as we will focus on those methods that we think are the most robust, simple to use and readily available to the research community. A more comprehensive review of all available techniques has been written by Olkhov-Mitsel and Bapat [1]. There are also several good review articles that cover particular methods in much more detail than is described here [2,3,4,5]. Additionally, there are specific web-based forums that can aid in the quest to find the most suitable method for analysis: epigenie.com [6] and http://www.protocol-online.org [7]. Furthermore, providers of fee-for service analysis can be found at https://www.scienceexchange.com [8] and https://genohub.com [9].

## 2. Profiling Whole Genome Methylation

Some broad examples of situations in which global genome methylation changes include [10]: (1) events that impact the DNA (de)methylation machinery [11,12]; (2) the treatment of cells with compounds, such as furan or azacytidine [13]; (3) cellular changes in brain tissue induced by learning [14] and epigenetic changes that contribute to tumorigenesis [15,16]. Section 1 will describe six methods by which such differences can be revealed (represented by Circle 1 in Figure 1).

### 2.1. HPLC-UV

The technique of HPLC-UV (high performance liquid chromatography-ultraviolet), developed by Kuo and colleagues in 1980 [17], is still considered to be the current “gold standard” assay for quantifying the amount of deoxycytidine (dC) and methylated cytosines (5 mC) present in a hydrolysed DNA sample. However, the utility of this method is significantly limited by the need for specialized laboratory equipment and the requirement of relatively large quantities (3–10 μg) of the DNA sample to be analysed. Briefly, the DNA must be hydrolysed into its constituent nucleoside bases, the 5 mC and dC bases separated chromatographically and, then, the fractions measured. Then, the 5 mC/dC ratio can be calculated for each sample, and this can be compared between the experimental and control samples.

### 2.2. LC-MS/MS

Liquid chromatography coupled with tandem mass spectrometry (LC-MS/MS) is an alternative high-sensitivity approach to HPLC-UV, which requires much smaller quantities of the hydrolysed DNA sample. In the case of mammalian DNA, of which ~2%–5% of all cytosine residues are methylated, LC-MS/MS has been validated for detecting levels of methylation levels ranging from 0.05%–10%, and it can confidently detect differences between samples as small as ~0.25% of the total cytosine residues [18], which corresponds to ~5% differences in global DNA methylation.

The procedure routinely requires 50–100 ng of DNA sample, although much smaller amounts (as low as 5 ng) have been successfully profiled [18,19,20,21]. Another major benefit of this method is that it is not adversely affected by poor-quality DNA (e.g., DNA derived from FFPE samples). However, the necessary expertise and equipment is not particularly widespread, and so it is not a commonly-used method to analyse DNA methylation. However, it might be a consideration if one has access to LC-MS/MS as a fee-for-service at specialized centres or through collaboration with the laboratories that possess such equipment and expertise (e.g., Zymo Research or Millis Scientific).

### 2.3. ELISA-Based Methods

There are several commercially available kits, all enzyme-linked immunosorbent assay (ELISA) based, that enable the quick assessment of DNA methylation status (listed in Table 1). ELISA-based assays are typically prone to high variability; thus, they are only suitable for the rough estimation of DNA methylation. Still, they are quick and easy to perform methods that serve well for the identification of large changes in global DNA methylation.

**Table 1:** ELISA-based kits for global DNA methylation profiling.

Company	Name of the Kit	Amount of DNA Required	Number of Citations
Cell Biolabs	Global DNA Methylation ELISA	2 μg	1 citation
Sigma-Aldrich	Imprint Methylated DNA Quantification kit (sandwich ELISA)	100 ng	14 citations
abcam	EpiSeeker methylated DNA Quantification Kit	100 ng	None
Active Motif	Global DNA Methylation Assay — LINE-1	1 μg	New method
Zymo Research	5-mC DNA ELISA Kit	100 ng	8 citations
Epigentek	MethylFlash Methylated DNA5-mC Quantification Kit (Colorimetric)	100 ng	88 citations
	MethylFlash Methylated DNA5-mC Quantification Kit (Fluorometric)	100 ng	9 citations

Briefly, the DNA sample is captured on an ELISA plate, and the methylated cytosines are detected through sequential incubations steps with: (1) a primary antibody raised against 5 Mc; (2) a labelled secondary antibody; and then (3) colorimetric/fluorometric detection reagents. As an example, the manufacturer Epigentek claims that their kits possess a discriminating power of 1:1000 when comparing between methylated and unmethylated DNA. Still, only relatively big changes in DNA methylation (~1.5–2 times) can be resolved by this method due to the high level of inter- and intra-assay variability [22].

The Global DNA Methylation Assay — LINE-1 from Active Motif is slightly different from other ELISA-based kits. It specifically determines the methylation levels of LINE-1 (long interspersed nuclear elements-1) retrotransposons, of which ~17% of the human genome is composed. These are well established as a surrogate for global DNA methylation [23,24,25]. Briefly, fragmented DNA is hybridized to biotinylated LINE-1 probes, which are then subsequently immobilized to a streptavidin-coated plate. Following washing and blocking steps, methylated cytosines are quantified using an anti-5 mC antibody, HRP-conjugated secondary antibody and chemiluminescent detection reagents. Samples are quantified against a standard curve generated from standards with known LINE-1 methylation levels. The manufacturers claim the assay can detect DNA methylation levels as low as 0.5%. Thus, by analysing a fraction of the genome, it is possible to achieve better accuracy in quantification.

### 2.4. LINE-1 + Pyrosequencing

Levels of LINE-1 methylation can alternatively be assessed by another method that involves the bisulfite conversion of DNA (discussed in detail in Section 2.1), followed by the PCR amplification of LINE-1 conservative sequences. The methylation status of the amplified fragments is then quantified by pyrosequencing, which is able to resolve differences between DNA samples as small as ~5% [24]. Even though the technique assesses LINE-1 elements and therefore relatively few CpG sites, this has been shown to reflect global DNA methylation changes very well. The method is particularly well suited for high throughput analysis of cancer samples, where hypomethylation is very often associated with poor prognosis [26,27,28]. This method is particularly suitable for human DNA, but there are also versions adapted to rat and mouse genomes. Furthermore, it is worth noting that data analysis can be outsourced to the company EpigenDx.

### 2.5. AFLP and RFLP

Detection of fragments that are differentially methylated could be achieved by traditional PCR-based amplification fragment length polymorphism (AFLP) [29], restriction fragment length polymorphism (RFLP) [30] or protocols that employ a combination of both [31,32,33].

In general, these methods are becoming extinct following the emergence of more powerful modern techniques. Their major limitation has always been that they can only assess a small percentage of global DNA methylation. Secondly, technical issues, such as achieving good resolution of multiple DNA bands, can be an issue that often hampers such techniques.

However, all three of the methods mentioned above (ELISA, AFLP and RFLP) are inexpensive ways to quickly assess DNA methylation. An additional advantage is that these methods could be used for any species, even with limited or no information about their DNA sequence composition. The methods of AFLP and RFLP can also be used for the isolation of differentially-methylated sequences, via their fractionation and subsequent extraction from the polyacrylamide gel.

### 2.6. LUMA

The LUMA (luminometric methylation assay) technique was published by Karimi and colleagues in 2006 [34]. It utilizes a combination of two DNA restriction digest reactions performed in parallel and subsequent pyrosequencing reactions to fill-in the protruding ends of the digested DNA strands. One digestion reaction is performed with the CpG methylation-sensitive enzyme HpaII; while the parallel reaction uses the methylation-insensitive enzyme MspI, which will cut at all CCGG sites. The enzyme EcoRI is included in both reactions as an internal control. Both MspI and HpaII generate 5′-CG overhangs after DNA cleavage, whereas EcoRI produces 5′-AATT overhangs, which are then filled in with the subsequent pyrosequencing-based extension assay. Essentially, the measured light signal calculated as the HpaII/MspI ratio is proportional to the amount of unmethylated DNA present in the sample. As the sequence of nucleotides that are added in pyrosequencing reaction is known, the specificity of the method is very high and the variability is low, which is essential for the detection of small changes in global methylation. LUMA requires only a relatively small amount of DNA (250–500 ng), demonstrates little variability and has the benefit of an internal control to account for variability in the amount of DNA input. However, high quality DNA is essential to ensure that complete enzymatic digestion occurs, and the polymerase extension assay requires a pyrosequencing machine and reagents.

### 2.7. Which Method to Use for Whole Genome Methylation Profiling?

It is important to note that all of the methods described above possess a tendency to either under or overestimate the amount of global DNA methylation present in a particular sample. In 2013, Lisanti and colleagues conducted a comparative study of several methods that included LINE-1, LUMA and HPLC. They concluded that of these assays, only the data generated from the LINE-1 assay correlated well with the “gold-standard” HPLC-derived measurements [35].

Prioritizing those methods that demonstrate the highest specificity, sensitivity and minimal assay-to-assay variability and also taking into account the amount starting material each assay requires, we would recommend, in order of preference: (1) LINE-1/pyrosequencing; (2) LC-MS/MS; and then (3) LUMA.

## 3. Identification of Differentially-Methylated Regions

The methods described in this review up until this point (represented by Circle 1 in Figure 1) can be used to determine the overall changes in the DNA methylation status of the sample(s) being analysed. However, how does one identify and assess specific genes/regulatory regions of interest that are differentially methylated? Section 3 will discuss the methods that are suited to this particular task (Circle 2 in Figure 1). All methods for identification of differentially methylated regions as well as all other methods describedin this paper are compared in Table 2.

### 3.1. Bisulfite Sequencing

The technique of bisulfite sequencing is considered to be the “gold standard” method in DNA methylation studies. Current DNA sequencing technologies do not possess the ability to distinguish methylcytosine from cytosine. The bisulfite treatment of DNA mediates the deamination of cytosine into uracil, and these converted residues will be read as thymine, as determined by PCR-amplification and subsequent Sanger sequencing analysis. However, 5 mC residues are resistant to this conversion and, so, will remain read as cytosine. Thus, comparing the Sanger sequencing read from an untreated DNA sample to the same sample following bisulfite treatment enables the detection of the methylated cytosines. With the advent of next-generation sequencing (NGS) technology, this approach can be extended to DNA methylation analysis across an entire genome.

Use of bisulfite sequencing can be challenging. Bisulfite conversion reduces genome complexity to three nucleotides (except the relatively rare 5 mC), and thus, post-NGS sequence alignment becomes a more difficult task. In addition, bisulfite conversion leads to DNA fragmentation, which, together with decreased complexity, makes amplification of long fragments difficult and could potentially result in the generation of chimeric products.

It is crucial to ensure complete conversion of non-methylated cytosines, as the estimated level of DNA methylation depends on it. Therefore, it is important to incorporate controls for bisulfite reactions, as well as to pay attention to the appearance of cytosines in non-CpG sites after sequencing, which is an indicator of incomplete conversion. Careful interpretation of DNA methylation level should take into consideration the homogeneity of the cell population, as the resulting ratio is a snapshot of all DNA isolated from the sample. A mixed population of cells with varying methylation status (e.g., cancer samples or tissues composed of mixed cell populations) will have a dilution effect and therefore leverage detected methylation level. An alignment problem could be lessened once we move from whole genome bisulfite sequencing to a subpopulation of methylated DNA. For an overview of the difficulties related to bisulfite sequencing and ways to overcome them, see [36].

Whole genome bisulfite sequencing (WGBS) is similar to whole genome sequencing, except for one detail: bisulfite conversion. It is the most comprehensive of all existing methods. The only limitations are the cost and difficulties in the analysis of NGS data. As already mentioned above, non-methylated cytosines become thymines after bisulfite treatment, and the DNA composed of just three bases is very difficult to assemble. Another limitation that existed until recently is that a considerable amount of DNA was required for WGBS, but modification of the protocol that postponed the adaptor ligation step till after bisulfite treatment allowed performing WGBS routinely from ~30 ng of DNA and, in some cases, even from as little as 125 pg [37]. However, since only a small fraction of the genome has the potential to be differentially methylated, WGBS is normally not required. Sequencing of the 5 mC-enriched fraction of the genome is not only a less expensive approach, but it also allows one to increase the sequencing coverage and, therefore, precision in revealing differentially-methylated regions. Methods for such an enrichment are discussed in Section 5. Sequencing could be done using any existing NGS platform; Illumina and Life Technologies both offer kits for such analysis.

**Table 2:** Comparison of methods for DNA methylation analysis.

Method	Availability as a Kit	Coverage	Sensitivity	Specificity	Amount of Starting Material	^§^ Price	References
**Methods for Whole Genome Profiling**			**Whole Genome Assessment without Identification of Differentially-Methylated Regions**				
Mass spectrometry (LC-MS/MS)	No	Whole genome assessment	*** detect 5% difference in methylation between samples	***	100 ng–1 μg	$80/sample (Millis Scientific)	[18,19,20,21]
LUMA (Luminometric-Based Assay)	No	Whole genome assessment	**	**	250–500 ng		[34,38]
LINE-1 + pyrosequencing	No	17% of the whole genome	***	**	50 ng		[39]
HPLC-UV	No	Whole genome assessment	*	**	3–10 μg		[17]
**Methods for Identification of Differentially-Methylated Regions**							
^#^ Enrichment of 5metC regions by pulldown with MBD protein (needs to be followed by NGS or microarray)	MethylCap kit (Diagenode)	~80% of all 5–5 mCpG	***	***	200 ng–1.2 μg	$550/48 rxns	[3,40]
	MethylMiner Methylated DNA Enrichment Kit (Thermo Fisher Scientific)		*	*	5 ng–1 μg	$485/25 rxns	
^#^ Enrichment for 5 mC regions by MBD2b/MBD3L1 proteins pulldown (MIRA-based assay) (needs to be followed by NGS or microarray)	MethylCollector Ultra (Active Motif)	No info	**	**	1 ng–1 μg	$495/10 rxns	[40,41]
^#^ Enrichment for 5 mC regions with antibodies (MeDIP) (needs to be followed by NGS)	MeDIP (Active Motif)	No info	No info	No info	100 ng–1 μg	$395/10 rxns	[42,43]
	MagMeDIP (Diagenode)	No info	No info	No info	1.2 μg	$595/48 rxns; $325/10 rxns	[44]
^#^ Enrichment for CpG rich regions with RNA baits (needs to be followed by NGS)	SureSelect Human Methyl-Seq (Agilent)	3.7 × 10^6^ CpGs	***	***	3 μg	~$320 (baits); +$70 (library prep)	[5]
^#^ Enrichment for CpG rich regions by hybridisation with bait oligonucleotides (needs to be followed by NGS)	SeqCap Epi CpGiant Enrichment Kit (Roche)	5.5 × 10^6^ CpGs	***	***	1 μg	~$450; + bisulfite conversion kit	[45,46]
HumanMethylation450 BeadChip array	Illumina	482.000 CpG sites (99% of known genes)	***	***	0.5–1 μg	~$9000/24 samples (2 chips)	[47]
Sequencing of methylation-enriched fraction of the genome (after MeDIP or MIRA)	Illumina	varies, depending on the sample	varies, as number of reads correlates with the amount of DNA	Depends on the enrichment method	> 50 ng	$360/sample	
Whole genome bisulfite sequencing (WGBS)	Illumina	100%		***	50–100 ng	$6000/sample	[48,49]
Reduced representation bisulfite sequencing (RRBS)	Illumina	~60% of promoters, 85% of CGI (~1.5 × 10^6^ CpGs)	varies, as sequencing depth correlates with the amount of DNA (20X coverage is recommended)	***	1 μg	$2700–5000/sample (sequencing and library prep)	[50,51]
Methyl-MiniSeq (improved RRBS)	Zymoresearch	>85% coverage of all CpG islands and >80% of all gene promoters (~3 × 10^6^ CpGs)	varies, as sequencing depth correlates with the amount of DNA (20X coverage is recommended)		100 ng–5 μg	$2800 (all steps and bioinformatics included)	[44,52]
Methyl-sensitive cut counting (MSCC) (needs to be followed by NGS)	No	1% of all CpG	depends on the sequencing coverage	***	1–5 μg		[53]
Improved MSCC (needs to be followed by NGS)	No	30% of CpGs (~58% of CpG-rich regions)		***	1–5 μg		[54,55]
**Low Throughput Methods**							
PCR-based (digestion followed by PCR)	OneStep qMethyl Kit (Zymoresearch)	Gene-specific 22 samples/ 96-well plate	***	***	≥20 ng		[56]
	EpiTect II DNA methylation enzyme Kit (Qiagen)				1–4 μg		[57]

**^#^** Enriched fractions are normally used for NGS. ^§^ All prices are approximate and have been obtained during 2015. *** Defines the best sensitivity or specificity (*** > ** > *).

Both limitations of WGBS are alleviated in reduced representation bisulfite sequencing (RRBS), where only a fraction of the genome is sequenced [50,51,58]. In RRBS, enrichment of CpG-rich regions is achieved by isolation of short fragments after MspI digestion that recognizes CCGG sites (and it cut both methylated and unmethylated sites). It ensures isolation of ~85% of CpG islands in the human genome. Then, the same bisulfite conversion and library preparation is performed as for WGBS. The RRBS procedure normally requires ~1 µg of DNA. It could be performed with only 100 ng of DNA, but it needs to be pure enough for successful MspI digestion. Amplification of bisulfite-treated DNA for NGS is not without problems; therefore, it is important to find the most recent procedure, such as in [58]. Enrichment for CpG-rich regions or specific regions of interest could be performed before NGS. Such enrichment could precede bisulfite conversion and be achieved by hybridization with immobilized oligonucleotides (so-called bait sequences). Such kits are commercially available (e.g., SureSelect Human Methyl-Seq from Agilent). Hybridization for enrichment could be done after bisulfite conversion using the SeqCap Epi CpGiant Enrichment Kit from Roche. Customized versions of these kits are available that allow enrichment for a small fraction of the genome that contains only the region(s) of interest. This approach is called targeted bisulfite sequencing. Both kits mentioned above show good correlation with RRBS, while covering more CpG-rich regions [59].

Less common is the detection of methylated bases directly through sequencing of unmodified DNA that could be done without enrichment or bisulfite conversion. Considering all of the disadvantages of bisulphate modifications, direct detection of modified bases would be a preferred approach. Pacific Biosciences company has developed a way to detect methylated bases directly by monitoring the kinetics of polymerase during single molecule sequencing and already offers a commercial product for such sequencing [60]. However, so far, the applicability of this technology for DNA methylation analysis was demonstrated with bacterial DNA only. There have been recent advances in the development of nanopore-based single-molecule real-time sequencing technology (SMRT), which is able to detect modified bases directly [61,62]. Commercialization of these new findings will bring the next generation of instruments with even better sensitivity and specificity.

### 3.2. Array or Bead Hybridization

Methylated DNA fractions of the genome, usually obtained by immunoprecipitation, could be used for hybridization with microarrays. Currently available examples of such arrays include: the Human CpG Island Microarray Kit (Agilent), the GeneChip Human Promoter 1.0R Array and the GeneChip Human Tiling 2.0R Array Set (Affymetrix). These arrays are approaches with good value for the money for pinpointing specific regions of interest, which can then be further interrogated by higher resolution methods.

The search for differentially-methylated regions using bisulfite-converted DNA (Circle 3 in Figure 1) could be done with the use of different techniques. Some of them are easier to perform and analyse than others, because only a fraction of the genome is used. The most pronounced functional effect of DNA methylation occurs within gene promoter regions, enhancer regulatory elements and 3′ untranslated regions (3′UTRs). Assays that focus on these specific regions, such as the Infinium HumanMethylation450 Bead Chip array by Illumina, can save time and money. The array can detect from ~500 ng of input DNA the methylation status of 485,000 individual CpG in 99% of known genes, including miRNA promoters, 5′ UTR, 3′ UTR, coding regions (~17 CpG per gene) and island shores (regions ~2 kb upstream of the CpG islands).

The experimental design is an adaptation of the Illumina GoldenGate high throughput single nucleotide polymorphism (SNP) system [63]. Briefly, bisulfite-treated genomic DNA is mixed with assay oligos, one of which is complimentary to uracil (converted from original unmethylated cytosine), and another is complimentary to the cytosine of the methylated (and therefore protected from conversion) site. Following hybridization, primers are extended and ligated to locus-specific oligos to create a template for universal PCR. Finally, labelled PCR primers are used to create detectable products that are immobilized to bar-coded beads, and the signal is measured. The ratio between two types of beads for each locus (individual CpG) is an indicator of its methylation level. The analysis of such an enormous amount of data has been the subject of the improvement that was published in [64,65,66]; though being aware that up to 6% of probes could give false positives due to cross-reactivity, as was recently demonstrated [67].

This is the most popular method for methylation profiling, which sits between whole genome bisulfite sequencing and low throughput methods that can access the methylation of a single locus. Over 360 publications to date used Illumina methylation arrays. According to Illumina, the price is about U.S. $300–360/sample. The minimum number of samples per kit is 24, and the chip for hybridization accommodates 12 samples, so it is still quite an expensive exercise for some labs and not suitable for small projects. Outsourcing such analysis to a big sequencing facility is a good option.

The chip is only suitable for the study of human genomic DNA. For scientist wishing to use this technique for non-human species, it is possible to use the Illumina instrument and kits with a custom panel, though this approach is limited to 384 CpG sites.

It is possible to purchase kits that exploit the extension of methylation-specific primers for validation studies. In the VeraCode Methylation assay from Illumina, 96 or 384 user-specified CpG loci are analysed with the GoldenGate Assay for Methylation. Differently from the BeadChip assay, the VeraCode assay requires the BeadXpress Reader for scanning.

### 3.3. Methyl-Sensitive Cut Counting: Endonuclease Digestion Followed by Sequencing

As an alternative to sequencing a substantial amount of methylated (or unmethylated) DNA, one could generate snippets from these regions and map them back to the genome after sequencing. Moreover, coverage in NGS could be good enough to quantify the methylation level for particular loci. The technique of serial analysis of gene expression (SAGE) has been adapted for this purpose and is known as methylation-specific digital karyotyping [68], as well as a similar technique, called methyl-sensitive cut counting (MSCC) [69,70]. Examples of its recent usage and some further modifications of the method can be viewed in the following papers [54,71,72,73].

**Figure 3:**
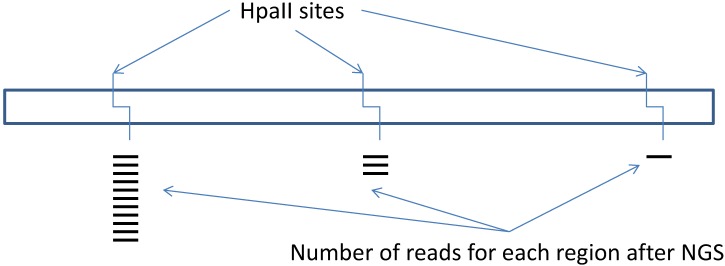
Scheme of the methyl-sensitive cut counting technique for elucidation of DNA methylation status for a particular gene of interest.

In summary, in all of these methods, methylation-sensitive endonuclease(s), e.g., HpaII is used for initial digestion of genomic DNA in unmethylated sites followed by adaptor ligation that contains the site for another digestion enzyme that is cut outside of its recognized site, e.g., EcoP15I or MmeI. These ways, small fragments are generated that are located in close proximity to the original HpaII site. Then, NGS and mapping to the genome are performed. The number of reads for each HpaII site correlates with its methylation level (Figure 3). A minimum of 2 μg of highly pure genomic DNA is required for these techniques.

Recently, a number of restriction enzymes have been discovered that use methylated DNA as a substrate (methylation-dependent endonucleases). Most of them were discovered and are sold by SibEnzyme: BisI, BlsI, GlaI. GluI, KroI, MteI, PcsI, PkrI [74]. The unique ability of these enzymes to cut only methylated sites has been utilized in the method that achieved selective amplification of methylated DNA [75]. Three methylation-dependent endonucleases that are available from New England Biolabs (FspEI, MspJI and LpnPI) are type IIS enzymes that cut outside of the recognition site and, therefore, are able to generate snippets of 32bp around the fully-methylated recognition site that contains CpG. These short fragments could be sequences and aligned to the reference genome [76]. The number of reads obtained for each specific 32-bp fragment could be an indicator of its methylation level. Similarly, short fragments could be generated from methylated CpG islands with *Escherichia coli*’s methyl-specific endonuclease McrBC, which cuts DNA between two half-sites of (G/A) mC that are lying within 50 bp–3000 bp from each other. This is a very useful tool for isolation of methylated CpG islands that again can be combined with NGS. Being bisulfite-free, these three approaches have a great potential for quick whole genome methylome profiling. The con of these techniques is that high quality DNA is required for digestion.

## 4. Methylation Status of Specific Genes of Interest

The following section will discuss methods that can be used for analysing the DNA methylation of specific genes/regions of interest. Bisulfite conversion is still the first step for many downstream methods (Circle 3 of Figure 1).

### 4.1. Bead Array

VeraCode Methylation technology from Illumina (http://www.illumina.com/content/dam/illumina-marketing/documents/products/datasheets/datasheet_veracode_methylation.pdf) [77] uses the same methodology as the HumanMethylation450 BeadChip array described in Section 2.2. It is an extension-based assay, which can be customized to profile up to 384 individual CpG sites, and unlike the HumanMethylation450 BeadChip array, it can be used to study non-human species.

### 4.2. PCR and Sequencing

**Figure 4:**
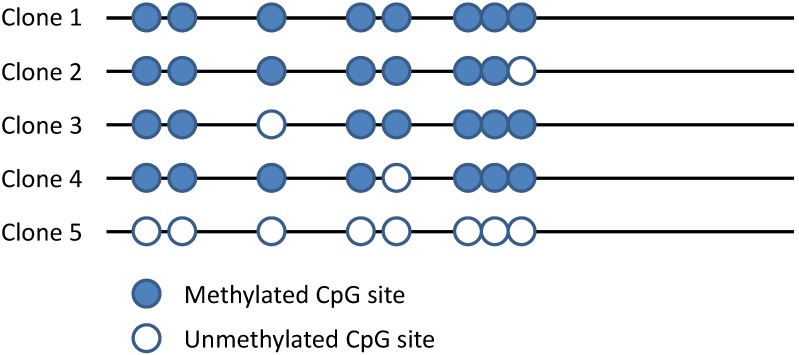
Typical presentation of the results for DNA methylation after bisulfite sequencing. The PCR product is cloned, and several randomly-selected plasmids are sequenced.

Bisulfite-converted DNA could be used for the amplification of the region of interest followed by sequencing. Primers are designed around the CpG island (using MethPrimer software at http://www.urogene.org/methprimer [78]) and used for PCR amplification of bisulfite-converted DNA. The resulting PCR products could be cloned and sequenced. Until recently, this was the only way to demonstrate the methylation status of individual CpG sites within the CpG island of interest. Sequencing results from several independent clones are presented as a beads-on-a-string picture (Figure 4). In some cases, when differences in methylation between samples are large (>50%), direct sequencing of the PCR product is an alternative [79]. Limitations of the method: nested PCR is often required in order to overcome the problem of unspecific amplification; primer design and amplification are often problematic due to the reduced complexity of DNA; and amplification of long fragments from bisulfite-treated DNA is difficult (the limit is 100–300 bp in most cases).

### 4.3. Pyrosequencing

Pyrosequencing is another technology suitable for low throughput projects [80]. Individual primers are designed or purchased as a kit (for example, PyroMark CpG Assays from Qiagen). PCR products are obtained, and short-read pyrosequencing reaction (~100 bp) is performed. The level of methylation for each CpG site within the sequenced region is estimated based on the signal intensities for incorporated dGTP and dATP. The result is quantitative, and the technique is able to detect even small differences in methylation (down to 5%). It is a good technique for heterogeneous samples (e.g., cancer), where only a fraction of cells has a differentially-methylated gene of interest. Pyrosequencing requires specialized equipment, such as PyroMark from Qiagen or the Qseq instrument from Bio Molecular Systems.

### 4.4. Methylation-Specific PCR

Another classical method that uses bisulfite-converted DNA is methylation-specific PCR [81]. To perform it, two pairs of primers are designed; one pair that favours amplification of methylated and another one of unmethylated DNA. Two qPCR reactions are performed for each sample, and relative methylation is calculated based on the difference of their Ct values. It is a quick method, which could be used for simultaneously profiling of multiple samples. The obvious drawback is that methylation status of only one or two CpG sites is assessed at a time. The program for the design of methylation-specific primers can be found at http://www.urogene.org/methprimer [78].

### 4.5. PCR with High Resolution Melting

The amplified PCR product could be analysed using high resolution melting. Most of the qPCR instruments have such a capability or special instruments could be used. Intercalating dye, e.g., SYBR or Eva green, when bound to double-stranded DNA is highly fluorescent, but as the temperature rises, the DNA strands dissociates, the dye goes back to solution and the fluorescence drops. DNA that was originally methylated retains cytosines and has a higher melting temperature compared to an unmethylated one. The level of methylation is correlated with the melting profile of the product. The drawback of the method is the necessity to obtain a pure PCR product, which is difficult in many cases. However, if a pure PCR product is obtained, then even small differences (5%–10%) in DNA methylation or the contribution of methylated DNA from subpopulations of cells within heterogeneous populations, such as cancer sample, could be detected [82].

### 4.6. COLD-PCR for the Detection of Unmethylated Islands

COLD-PCR is able to detect rare unmethylated sequences among an excess of methylated copies [83]. It is a very sensitive method used for diagnostic purposes. CpG islands heavily methylated in normal tissues often become hypomethylated in cancer, and the ability to detect them is important in clinical practice. The first step is bisulfite conversion. The principle of COLD-PCR is simple: the denaturation temperature of the PCR is lowered to allow only unmethylated (and therefore less GC-rich) fragments to be efficiently amplified. Obviously, to be efficient, target sequences should contain several CpG sites.

Addressing the same question of the analysis for heterogeneous samples, methylation-specific electrophoresis was developed [84]. Currently, it requires the use of denaturing gradient polyacrylamide, but in the future, other technologies, such as DHPLS, will make this method less labour intensive. The sensitivity of the method allowed detecting methylated DNA at a level of 0.1% in the mix.

## 5. Digestion-Based Assays

There are some methods that do not need bisulfite conversion; they are based on the selective digestion of DNA by particular endonucleases (Circle 4 in Figure 1). Historically, this was actually the first approach utilized by biologists in the study of DNA methylation [85]. The endonuclease HpaI is able to digest the CCGG sequence, but only when it is un-methylated, In contrast, the MspI enzyme, which also cuts DNA at CCGG sites, is unaffected by DNA methylation. Hence, the digestion of the same DNA sample with HspI and MspI, and electrophoretic analysis of the size of the digestion products, can reveal the location of the sites of DNA methylation. This approach has also been adapted to a method for whole genome scanning [86].

Nowadays, there are a number of methods that incorporate such digestion. Some of them are suitable for whole genome methylation profiling (Circles 3 and 4 in Figure 1), while others are designed in a way to estimate the methylation level of individual genes (Circle 1 in Figure 1).

Methylation analysis of particular human or rodent gene promoters can be performed using Qiagen’s EpiTect Methyl II PCR Array System (Circle 1 on Figure 1) [57]. This kit represents the most recent step in the perfection of traditional digestion-based methods that utilize pairs of methylation-sensitive and methylation-insensitive enzymes. Briefly, a DNA sample is split into three tubes, each containing a different endonuclease activity that preferentially cuts: (1) methylated DNA; (2) un-methylated DNA; and (3) both methylated and un-methylated DNA. In a fourth tube, the DNA remains undigested. Quantitative-PCR is used to estimate the methylation level of a particular CpG island/promoter, and specific primers are included as part of the kit. A simple spreadsheet software program is provided as part of the kit, which uses the qPCR data to calculate the percentage of the DNA sample that is methylated. Unfortunately, the exact length of the amplified CpG island/promoter and the physical location of the annealing sites of the PCR primers are not disclosed. Furthermore, methylation cannot be assessed for genes without defined CpG islands. Among the advantages are the streamlined kit-type format and the possibility to order off-the-shelf primers for specific genes of interests.

The less expensive home-made “old but gold” approach uses digestion with methylation-sensitive enzyme followed by qPCR with primers that surround the cutting site (there are a number of enzymes to choose from: HpaII, AatII, ClaI, *etc.*). DNA fragmentation (e.g., sonication) should be performed before digestion. DNA input could be measured by the amplification of an unrelated fragment that is unaffected by digestion. Digestion efficiency needs to be controlled also to ensure that it is close to completion in all samples [79]. It is possible to buy kits for such analysis: the OneStep qMethyl kit from Zymo Research that claims to be effective with just 20 ng of purified DNA.

We have already discussed the use of methylation-dependent endonucleases in the generation of short fragments from heavily-methylated regions of the genome. The same enzymes could be used for digestion followed by PCR. This way, the same region of interest could be assessed side by side with two types of enzymes: methylation sensitive and methylation dependent.

## 6. Enrichment Strategies in Search of Differentially-Methylated Loci

Sequencing of a subpopulation of DNA could decrease the amount of sequencing and consequently the price, as well as ease the bioinformatics of the project. There are two major strategies for such enrichment: pull-down of CpG-rich regions and hybridization with specific bait probes.

Pull-down of methylated DNA is possible with anti-methylcytosine binding proteins (MBD) or antibodies against 5 mC (MeDIP). Once obtained from different samples, methylated fractions of DNA could be directly compared using microarray hybridization or used for NGS. An approach that combined MeDIP and bisulfite conversion has been recently published; it has an improved level of specificity and provided single-CpG resolution [87].

The difference between antibody-based and MBD protein-based enrichment is that antibodies work better with single-stranded (denatured) DNA, while MBD proteins bind dsDNA. MBD methods tend to achieve slightly better enrichment of CpG islands, while MeDIP provides superior enrichment of genomic regions with low CpG density. However, both methods have been found to be 99% concordant (their difference did not exceed a given threshold), when assessing methylation levels at CpGs and non-CpGs cytosines with NGS platforms [88]. A very high level of specificity toward methylated DNA is achieved by the use of a MBD2b and MBD3L1 mix (the so-called MIRA method) [89]. Examples of MBD-based kits are: the MethylCap kit (Diagenode), MethylCollector Ultra kit (Active Motif), MethylMiner (Life Technologies) and CpG MethylQuest DNA Isolation kit (EMD Millipore). According to a recent comparison of several kits, the MethylCap kit (Diagenode) and MethylCollector Ultra kit (Active Motif) demonstrate the best combination of sensitivity and specificity [40]. Examples of MeDIP kits include: the Methylated-DNA IP Kit (Zymo Research), MagMeDIP Kit (Diagenode) and Methylamp Methylated DNA Capture (MeDIP) Kit (Epigentek).

A comparison of several techniques for enrichment [90] before NGS has demonstrated that both MeDIP- and MBD-based methods are inferior to restriction-based methods, such as reduced representation bisulfite sequencing (RRBS) [51] and HELP-seq/Methyl-Seq [91,92]. However, in contrast to restriction-based methods, immunoprecipitation does not require high quality DNA and provides better coverage, as it is not limited to MspI or other enzymes’ recognition sites.

In some cases, hypomethylated regions are of interest, and therefore, isolation of such regions would complement the analysis of the 5 mC-enriched fraction of the genome. Several methods are addressing this problem. The HypoMethyl collector kit from Active Motif is based on the active binding of unmethylated CpG islands by the CXXC binding domain from mouse Mbd1 protein.

The subtraction of undesired repetitive sequences (that make ~50% of the human genome) before bisulfite conversion could represent another form of enrichment, this time for unique sequences (such a service is available from Evrogene).

Positive enrichment through hybridization oligonucleotide baits for desired regions of interest, also known as targeted methylome sequencing, is another way of selecting regions that are more likely to be differentially methylated [93,94,95,96]. SureSelect Human Methyl-Seq from Agilent is a commercially available kit for targeted methylome enrichment. In this method, CpG islands and some other fragments with CpGs (altogether 3.7 million CpGs) are enriched through an in-solution hybridization protocol. Later on, these fragments are bisulfite-converted and used for NGS. The idea is that such enrichment for CpG-containing regions before bisulfite conversion is independent of and therefore unbiased by their methylation status and simultaneously helps to decrease the complexity of the DNA pool before sequencing.

Enrichment for specific regions after bisulfite conversion is also possible, e.g., with the SeqCap Epi CpGiant Enrichment Kit (Roche). In this case, DNA is first fragmented and ligated with adaptors for NGS, then bisulfite converted and, only after that, hybridized with RNA baits designed for CpG-rich regions of the genome. It targets ~5.5 million CpGs. Besides the main kit designed for the human genome, Roche also offers custom versions of the kit to perform enrichment for specific regions of interest or for non-human DNA.

Theoretically enrichment for specific sequences before bisulfite treatment is less biased than the one performed after bisulfite conversion. In reality, both methods demonstrated similar performances in revealing differentially-methylated regions and are concordant with RRBS and Illumina’s 450 K array [45]. Roche’s enrichment strategy could be slightly advantageous, as it is designed to pick up all SNP variants. Both kits are targeting gene enhancers, which are not present in Illumina’s 450K array.

Finally, there is a method designed to obtain both methylated and unmethylated fractions of DNA from the same sample [97]. Short fragments of unmethylated DNA are collected after digestion with methylation-sensitive restriction enzyme(s), whilst longer methylated loci are concatemerized, amplified with phi29 polymerase and subsequently digested with the same restriction enzyme to obtain a fraction of methylated DNA. Amplification of any remaining unmethylated fragments is prevented by the use of blocking adaptors.

## 7. Detection of Hydroxymethyl Cytosine

Finally, we would like to briefly describe methods that aim to detect 5-hydroxymethylcytosine (5-hmC) and distinguish it from 5 mC. Essentially, there are two types of approaches for which this can be achieved: digestion- and antibody-based techniques.

It has been determined that the enzyme glucosyltransferase will modify 5-hmC, but not 5 mC bases. Based on this intrinsic difference in their properties as substrates, both New England Biolabs and Zymo Research have developed assay kits for the specific detection of glycosylated 5-hmC.

Zymo Research’s Quest hmC Detection Kit utilizes hydroxymethylcytosine glucosyltransferase and the substrate uridine diphosphoglucose to selectively glucosylate 5-hmC bases. Importantly, modification of the 5-hmC bases renders these sites of the DNA strand resistant to cleavage by the nuclease MspI. Digested DNA is then analysed by either NGS or the qPCR assay. The EpiMark 5-hmC and 5-mC Analysis Kit by New England Biolabs uses the action of T4 beta-glucosyltransferase, followed by MspI digestion to accomplish the same task.

Enrichment for 5-hmC-containing DNA could be done with anti-5-hmC antibodies. They are available from several companies.

## 8. Conclusions

In our opinion, the methods that will gain the most popularity are those that are commercially available in an easy-to-use kit format or that are not too technically demanding and that require equipment that is readily available at most academic institutions. NGS is rapidly becoming a more affordable option, and it is inevitable that this will become the standard technology upon which all global epigenetic profiling is based. Indeed, the use of molecular tools, such as methylation-specific digital karyotyping (or similar technique), will become commonplace. However, validation of NGS datasets, within specific subsets of genes, will still be achieved through pyrosequencing and methylation-specific PCR. Further down the track, technology enabling the direct detection of modified bases within individual molecules of DNA [60,61,62] will ultimately be the future of all DNA methylation studies.
